# From Natural Compounds to Health Innovation: Phytochemical Profiles, Biological Mechanisms, and Functional Potential of Plant-Derived Molecules in Food and Herbal Applications

**DOI:** 10.3390/plants15020186

**Published:** 2026-01-07

**Authors:** Dario Donno

**Affiliations:** Dipartimento di Scienze Agrarie, Forestali e Alimentari, Università di Torino, Grugliasco, 10095 Turin, Italy; dario.donno@unito.it

## 1. Introduction

For centuries, plants have been central in human medicine, food/nutrition, and culture. In the scientific community, their complex matrices—including bioactive molecules, phytochemicals, peptides, nutritional substances, and health-promoting metabolites—inspire researchers and technicians to improve human nutrition and health and develop sustainable, innovative solutions in pharmaceutical/herbal and food systems [1]. As interest in functional foods, herbal products, plant-based foods, and nutraceuticals has increased due to their potential health-promoting properties, there is an important need for multidisciplinary studies in order to connect phytochemical composition and relative biological mechanisms with practical applications and processing technologies [2]. The nutritional profile of plant-derived foods included fiber, micro- and macronutrients, mineral elements, and vitamins, contributing to overall well-being. Moreover, some classes of phytomolecules, such as polyphenols, monoterpenes, and carotenoids, influence the anti-cancer activity, anti-inflammatory properties, and antioxidant capacity of these herbal products and plant-based foods [3].

The Special Issue “Phytochemical Profiles, Health-Promoting Potential, and Botanical Mechanisms of Plant-Derived Components for Use in Food and Herbal Applications” aimed to explore, describe, and characterize the bioactive components and nutrients in plant-based sources, herbal preparations, food applications, and natural food supplements, highlighting their potential roles in human health and disease prevention. This Special Issue also aimed to (i) evaluate and describe the main analytical techniques and methodologies used to extract, identify, and quantify the nutritional substances and bioactive components in plant-based foods and herbal supplements; (ii) define innovative strategies to better characterize these complex plant-based matrices; and (iii) highlight the importance of a multivariate and interdisciplinary approach to assess the health-positive or side effects derived from their consumption, particularly in cases of diabetes, inflammatory diseases, cancers, and cardiovascular disorders. Insights into the impact of processing methods on nutrient and molecule bioavailability have also been discussed and provided. This Special Issue of *Plants* was designed to address these needs.

The published manuscripts present scientific rigor and multidisciplinary research, ranging from the valorization of agro-industrial by-products and residues to in-depth investigations of plant-derived compounds and innovative encapsulation strategies, together with studies on cellular and in vivo disease models. These works provide information on strategies to analyze, transform, and apply plant components for human benefit.

## 2. Valorization of By-Products, Crop Remains, and Plant Resources

The main topic in this Special Issue was the sustainable utilization of plant materials, in particular, materials traditionally considered as by-products or waste. The manuscript “Freeze-Drying for the Reduction of Fruit and Vegetable Chain Losses: A Sustainable Solution to Produce Potential Health-Promoting Food Applications” by Donno et al. [4] includes this approach. The challenge of post-harvest losses is important in the horticultural sector; in this work, the authors evaluated a freeze-drying strategy to preserve the biological and chemical richness of agricultural residues and develop new health-promoting foods, with additional economic benefits for growers and farmers. Phytochemical screening showed that horticultural crop remains retained significant amounts of phenolic acids, flavonols, and total phenolics, confirming that the fruit and vegetable residual materials remained biologically active after processing. The antioxidant capacity (FRAP assays) presented significant reducing power among the considered matrices. Moreover, multivariate statistical analyses (Principal Component Analysis—PCA) highlighted the specific phytochemical profiles of different considered products, while bioinformatic tools for biological target prediction suggested potential molecular pathways influenced by these molecules. This study shows that freeze-dried products derived from horticultural crop remains may be considered sustainable, accessible, and health-promoting ingredients for food applications, aligning environmental protection with nutritional and health innovation.

Another valorization approach is presented in “Enhanced Oligopeptide and Free Tryptophan Release from Chickpea and Lentil Proteins: A Comparative Study of Enzymatic Modification with Bromelain, Ficin, and Papain”. In this study, Domokos-Szabolcsy et al. [5] focused on legumes as an excellent protein source with a significant role for plant-derived diets. The research systematically evaluated how several plant proteases affected the hydrolysis profiles of lentil and chickpea proteins. Based on controlled enzymatic modification, the authors showed that papain improved the release of free amino acids and small oligopeptides. In particular, this enzyme enhanced the release of tryptophan, a serotonin precursor with a potential role in metabolic health and mood regulation. The peptide fraction identification in a specific range (200–1000 Da) was especially important because these small peptides sometimes exhibit potential bioactivity, such as anti-inflammatory, antioxidant, or antihypertensive effects, together with better bioavailability. This paper enriched the industrial and scientific understanding of how enzymatic activity may be utilized to modify proteins from plant sources to develop advanced health-promoting and functional foods, targeted nutraceutical products, or protein supplements.

## 3. Delivery Systems for Bioactive Compounds and Technological Innovation

A key direction presented in this Special Issue is innovative formulation technologies to enhance the bioavailability, dispersion, and stability of plant-derived molecules. The manuscript “A Systematic Preparation of Liposomes with Yerba Mate (*Ilex paraguariensis*) Extract” by Micheletto et al. [6] investigated the encapsulation of an extract derived from yerba mate in small lipid vesicles; this system is a technological approach relevant to incorporating sensitive bioactive molecules into therapeutic formulations or food systems. The authors produced liposomal systems utilizing phosphatidylcholine and a reverse-phase evaporation, obtaining stable nanoscale vesicles. The results were confirmed by dynamic light scattering, zeta potential measurements, UV–Vis profiling, and FTIR spectroscopy. Their findings showed that interactions between phytochemicals and lipids may modify vesicle structure, potentially improving the controlled release and protection of yerba mate molecules. The outputs of this study may be considered an important advancement in the delivery technology application in functional foods, with promising capacities to integrate plant bioactive molecules into complex matrices without compromising their efficacy or stability.

## 4. Mechanistic Advances in Disease-Related Models

Beyond compositional and technological studies, two papers in this Special Issue significantly improve the understanding of biochemical mechanisms used by plant-derived molecules to exert health benefits in humans.

The research “Araçá-Boi Extract and Gallic Acid Reduce Cell Viability and Modify the Expression of Tumor Suppressor Genes and Genes Involved in Epigenetic Processes in Ovarian Cancer” by Borsoi et al. [7] provides a robust study into the anti-cancer capacity of *Eugenia stipitata* (araçá-boi). The authors chemically profiled an extract (composed of flavonoids, phenolics, and organic acids) and then evaluated its antioxidant properties. They assessed and tested its effects on ovarian cancer cell lines, showing dose-dependent cytotoxicity, together with a significant modulation of gene expression in relation to epigenetic regulation and tumor suppression. Even if no changes in BRCA1 promoter methylation are demonstrated, the results presented transcriptional modulation as a potential action mechanism. This research provided significant evidence on Amazonian fruit species as potential sources of bioactive molecules with an important role in oncological applications.

The paper “Antiarrhythmic Effects of Supercritical Extract of *Acmella oleracea* in Rats: Electrophysiological Evidence and Cardioprotective Potential” by de Souza e Silva et al. [8] extended the health-promoting purpose of plant-derived compounds into cardiovascular physiology. Using a spilanthol-rich extract obtained by supercritical CO_2_ extraction, the authors tested its impact on cardiac electrophysiology in rat models. Their results showed that the extract counteracted the arrhythmic events induced by epinephrine in a dose-dependent manner, preserved key ECG intervals, and stabilized the sinus rhythm. The extract potential, similar to standard antiarrhythmic pharmaceutical formulations for several parameters, strongly suggests that *Acmella oleracea* may be utilized as a basis to develop future natural drugs against arrhythmias. This research demonstrated that scientific in vivo experimental trials may confirm traditional ethnobotanical uses with well-controlled pharmacological evidence.

## 5. Conclusions and Future Directions

The studies presented in this Special Issue collectively show a multidisciplinary field in which food science, sustainability, biology, chemistry, and pharmacology synergistically interact. Several general trends emerged, such as (i) integration between phytochemistry and advanced analytical strategies allows for fingerprinting of bioactive molecules, with an easier correlation among function and composition; (ii) sustainable strategies for the valorization agri-food residues and horticultural remains may transform crop losses and by-products into health-promoting ingredients and support circular economy models in the agricultural and food sectors; (iii) formulation technologies and processing, such as liposomal encapsulation and enzymatic tailoring, are very important to protect sensitive phytocompounds and optimize bioavailability; and (iv) disease-focused and mechanistic restudies present translational insight, allowing plant-derived molecules to be evaluated in clinical contexts.

Significant progress is highlighted in the present Special Issue, but several critical issues and challenges remain for further consideration. The main limitation in the research on plant-based bioactives is the translation from in vitro compositional results into robust in vivo clinical evidence. Moreover, innovative analytical strategies and biological tests and screenings allow an excellent characterization of phytochemical levels and profiles, but a greater standardization of bioavailability assessments, extraction protocols, and dose–response relationships is necessary and essential to ensure applicability and reproducibility in the real world. Smart delivery systems and sustainable processing strategies should be developed together with safety and regulatory frameworks for the transition of plant-derived molecules from experimental studies to health-promoting and functional food and therapeutic applications.

These papers present the technological and scientific breadth required to fully study the health-promoting and functional potential of plant-derived compounds. Future research will integrate nutrition frameworks, advanced material sciences, and omics-based tools to further demonstrate how botanical and dietary compounds may influence human health status and physiology, elucidating synergistic interactions and molecular targets. This Special Issue may inspire future studies and investigations, stimulate new collaborations, and provide a strong base for continued improvements to evaluate plant-derived molecules for therapeutic, health, and food applications.

## References

[1] Donno D., Turrini F. (2020). Plant Foods and Underutilized Fruits as Source of Functional Food Ingredients: Chemical Composition, Quality Traits, and Biological Properties. Foods.

[2] Donno D. (2021). Screening, Identification, and Quantification of Nutritional Components and Phytochemicals in Foodstuffs. Foods.

[3] Essa M.M., Bishir M., Bhat A., Chidambaram S.B., Al-Balushi B., Hamdan H., Govindarajan N., Freidland R.P., Qoronfleh M.W. (2023). Functional foods and their impact on health. J. Food Sci. Technol..

[4] Donno D., Neirotti G., Fioccardi A., Razafindrakoto Z.R., Tombozara N., Mellano M.G., Beccaro G.L., Gamba G. (2025). Freeze-Drying for the Reduction of Fruit and Vegetable Chain Losses: A Sustainable Solution to Produce Potential Health-Promoting Food Applications. Plants.

[5] Domokos-Szabolcsy É., Alshaal T., Elhawat N., Kovács Z., Kaszás L., Béni Á., Kiss A. (2024). Enhanced Oligopeptide and Free Tryptophan Release from Chickpea and Lentil Proteins: A Comparative Study of Enzymatic Modification with Bromelain, Ficin, and Papain. Plants.

[6] Micheletto Y.M.S., Jesus B.V.d., Peres G.L., Pinto V.Z. (2025). A Systematic Preparation of Liposomes with Yerba Mate (*Ilex paraguariensis*) Extract. Plants.

[7] Borsoi F.T., Arruda H.S., Andrade A.C., Dos Santos M.P., da Silva I.N., Marson L.A., Saliba A.S.M.C., de Alencar S.M., Geraldo M.V., Neri Numa I.A. (2025). Araçá-Boi Extract and Gallic Acid Reduce Cell Viability and Modify the Expression of Tumor Suppressor Genes and Genes Involved in Epigenetic Processes in Ovarian Cancer. Plants.

[8] de Souza e Silva A.P., Pires F.C.S., Ferreira M.C.R., Siqueira L.M.M., Ortiz de Menezes E.G., de Carvalho M.E.F., Nascimento L.A.S.d., Santos A.S., Hamoy A.O., Hamoy M. (2025). Antiarrhythmic Effects of Supercritical Extract of *Acmella oleracea* in Rats: Electrophysiological Evidence and Cardioprotective Potential. Plants.

